# KRAS Mutation Is a Predictor of Oxaliplatin Sensitivity in Colon Cancer Cells

**DOI:** 10.1371/journal.pone.0050701

**Published:** 2012-11-28

**Authors:** Yu-Lin Lin, Jau-Yu Liau, Shan-Chi Yu, Da-Liang Ou, Liang-In Lin, Li-Hui Tseng, Yih-Leong Chang, Kun-Huei Yeh, Ann-Lii Cheng

**Affiliations:** 1 Department of Oncology, National Taiwan University Hospital, Taipei, Taiwan; 2 Department of Pathology, National Taiwan University Hospital, Taipei, Taiwan; 3 Department of Internal Medicine, National Taiwan University Hospital, Taipei, Taiwan; 4 Department of Laboratory Medicine, National Taiwan University Hospital, Taipei, Taiwan; 5 Department of Medical Genetics, National Taiwan University Hospital, Taipei, Taiwan; 6 National Center of Excellence for Clinical Trial and Research, National Taiwan University Hospital, Taipei, Taiwan; 7 Department of Clinical Laboratory Sciences and Medical Biotechnology, College of Medicine, National Taiwan University, Taipei, Taiwan; 8 Graduate Institute of Oncology, College of Medicine, National Taiwan University, Taipei, Taiwan; 9 Graduate Institute of Clinical Medicine, College of Medicine, National Taiwan University, Taipei, Taiwan; 10 Department and Graduate Institute of Pathology, College of Medicine, National Taiwan University, Taipei, Taiwan; Baylor University Medical Center, United States of America

## Abstract

Molecular biomarkers to determine the effectiveness of targeted therapies in cancer treatment have been widely adopted in colorectal cancer (CRC), but those to predict chemotherapy sensitivity remain poorly defined. We tested our hypothesis that KRAS mutation may be a predictor of oxaliplatin sensitivity in CRC. KRAS was knocked-down in KRAS-mutant CRC cells (DLD-1^G13D^ and SW480^G12V^) by small interfering RNAs (siRNA) and overexpressed in KRAS-wild-type CRC cells (COLO320DM) by KRAS-mutant vectors to generate paired CRC cells. These paired CRC cells were tested by oxaliplatin, irinotecan and 5FU to determine the change in drug sensitivity by MTT assay and flow cytometry. Reasons for sensitivity alteration were further determined by western blot and real-time quantitative reverse transcriptase polymerase chain reaction (qRT -PCR). In KRAS-wild-type CRC cells (COLO320DM), KRAS overexpression by mutant vectors caused excision repair cross-complementation group 1 (ERCC1) downregulation in protein and mRNA levels, and enhanced oxaliplatin sensitivity. In contrast, in KRAS-mutant CRC cells (DLD-1^G13D^ and SW480^G12V^), KRAS knocked-down by KRAS-siRNA led to ERCC1 upregulation and increased oxaliplatin resistance. The sensitivity of irinotecan and 5FU had not changed in the paired CRC cells. To validate ERCC1 as a predictor of sensitivity for oxaliplatin, ERCC1 was knocked-down by siRNA in KRAS-wild-type CRC cells, which restored oxaliplatin sensitivity. In contrast, ERCC1 was overexpressed by ERCC1-expressing vectors in KRAS-mutant CRC cells, and caused oxaliplatin resistance. Overall, our findings suggest that KRAS mutation is a predictor of oxaliplatin sensitivity in colon cancer cells by the mechanism of ERCC1 downregulation.

## Introduction

Biomarkers to determine treatment efficacy have been investigated in the traditional chemotherapy era, but only a limited number of biomarkers has been found thus far. Examples are excision repair cross-complementation group 1 (ERCC1) expression to predict the resistance of oxaliplatin [1], and thymidylate synthase (TS) expression to determine 5FU sensitivity [2]. This concept has evolved and has become more relevant while treatment has advanced to molecular-targeted era. Most molecular-targeted agents have predefined targets, which facilitate predicting the efficacy of the treatment or prognosis of diseases. Good examples are epidermal growth factor receptor (EGFR) mutation for predicting the effectiveness of EGFR tyrosine kinase inhibitors (TKIs) in lung adenocarcinoma [3], as well as KRAS mutation for predicting the unresponsiveness of EGFR monoclonal antibody in colorectal cancer (CRC) [4]. Although extensive studies have been undertaken to identify new predictors from known signaling pathways or microarray-based studies [5], [6], biomarkers to predict chemotherapy sensitivity remain poorly defined.

**Figure 1:**
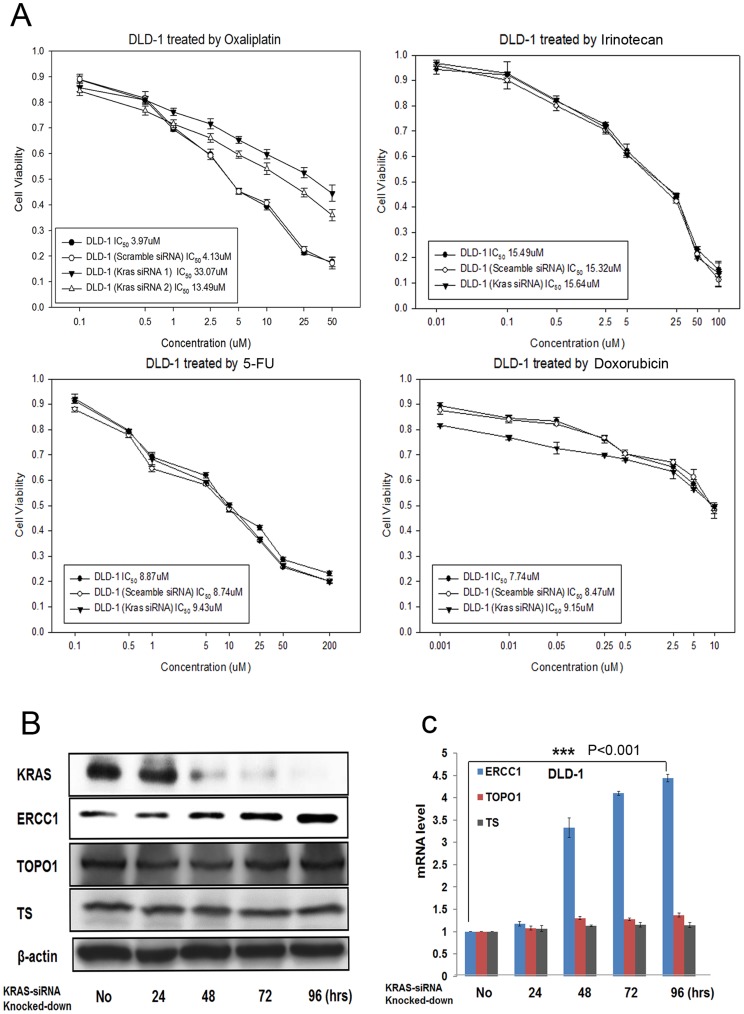
Knocking down KRAS expression in KRAS-mutant (G13D) CRC cells confers oxaliplatin resistance and ERCC1 upregulation. (A) KRAS-knocked-down DLD-1^G13D^ cells were more resistant to oxaliplatin, but have the same sensitivity to irinotecan, 5FU, and doxorubicin than parental DLD-1^G13D^ cells, as demonstrated by MTT assay. (B) The protein level of ERCC1, but not those of TOPO1 or TS, was upregulated after DLD-1^G13D^ cells were knocked-down by KRAS siRNA. (C) The mRNA level of ERCC1, but not those of TOPO1 or TS, was upregulated after DLD-1^G13D^ cells were knocked-down by KRAS siRNA. ***: *p*<0.001.

Several post hoc analyses of recent randomized trials on CRC suggested that the KRAS gene mutation status might predict the efficacy of cytotoxic chemotherapy, especially for oxaliplatin-based regimens. OPUS [7] and PRIME [8] studies, which were both designed for patients to receive first-line oxaliplatin/5FU/leucovorin with/without EGFR monoclonal antibodies, are good examples. Primarily focusing on the chemotherapy-only group in these 2 studies shows that first-line progression-free survival (PFS) in the KRAS mutant group lasted longer than that in the wild-type group, with 8.6 versus 7.2 months in the OPUS study, and 8.8 versus 8.0 months in the PRIME study. By contrast, in CRYSTAL study [9], which was designed for patients receiving first-line irinotecan/5FU/leucovorin with/without EGFR monoclonal antibody, a similar phenomenon was not observed. The median first-line PFS in KRAS-mutant and wild-type patients was 7.7 and 8.4 months, respectively.

According to these observations, we hypothesized that KRAS mutation may be a predictor of oxaliplatin sensitivity in CRC. First, KRAS was knocked-down in KRAS-mutant CRC cells and overexpressed in KRAS-wild-type CRC cells. These paired CRC cells were tested by oxaliplatin, irinotecan and 5FU to evaluate the change in drug sensitivity. Reasons for sensitivity alteration were further determined by western blot and real-time quantitative reverse transcriptase polymerase chain reaction (qRT -PCR). Finally, the target responsible for sensitivity alteration was validated by knocking-down and overexpressing the target.

**Figure 2:**
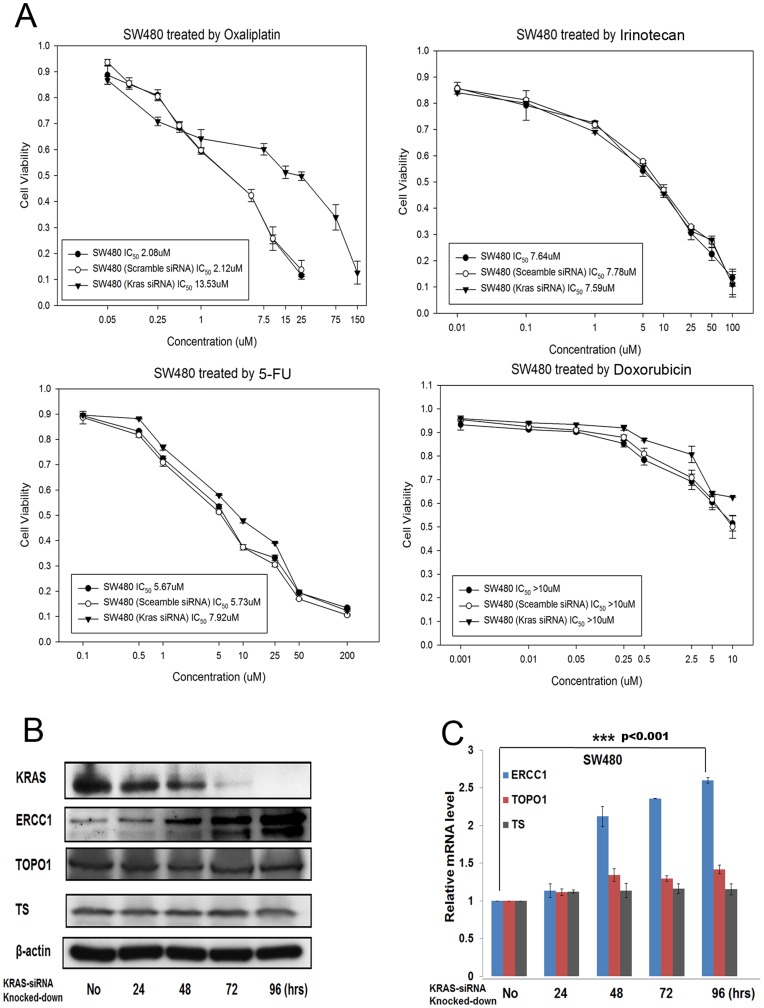
Knocking down KRAS expression in other KRAS-mutant subtype (G12V) CRC cells results in oxaliplatin resistance and ERCC1 upregulation. (A) KRAS-knocked-down SW480^G12V^ cells were more resistant to oxaliplatin, but have the same sensitivity to irinotecan, 5FU, and doxorubicin than parental SW480^G12V^ cells, as demonstrated by MTT assay. (B) The protein level of ERCC1, but not those of TOPO1 or TS, was upregulated after SW480^G12V^ cells were knocked-down by KRAS siRNA. (C) The mRNA level of ERCC1, but not those of TOPO1 or TS, was upregulated after SW480^G12V^ cells were knocked-down by KRAS siRNA. ***: *p*<0.001.

## Materials and Methods

### Cell Culture and Reagents

Human CRC cell lines COLO320DM (KRAS-wild-type), DLD-1^G13D^ (KRAS G13D mutation), and SW480^G12V^ (KRAS G12V mutation) were all obtained from American Type Culture Collection. Cells were all maintained in RPMI-1640 containing 10% fetal bovine serum, 2 mmol/L of L-glutamine (Life Technologies, Carlsbad, CA, USA), and PSA (10,000 units/ml of penicillin, 10 mg/ml of streptomycin, and 25 µg/ml amphotericin B; Biological Industries, Kibbutz Beit Haemek, Israel) and cultured at 37°C in a humidified incubator containing 5% CO2. Oxaliplatin (Eloxatin® injection 5 mg/ml) was obtained from Sanofi-Aventis Co., Ltd. (Taipei, Taiwan). Irinotecan, 5FU, and doxorubicin were all purchased from Sigma-Aldrich (St. Louis, MO, USA). Rabbit antibodies for western blot against ERCC1 and KRAS were purchased from Cell Signaling Technology, Inc. (Beverly, MA, USA). Mouse antibodies against TS, topoisomerase I (TOPO I), and β-actin were obtained from Millipore (Bedford, MA, USA), BD Biosciences (San Jose, CA, USA) and Cell Biolabs, Inc. (San Diego, CA, USA), respectively.

**Figure 3:**
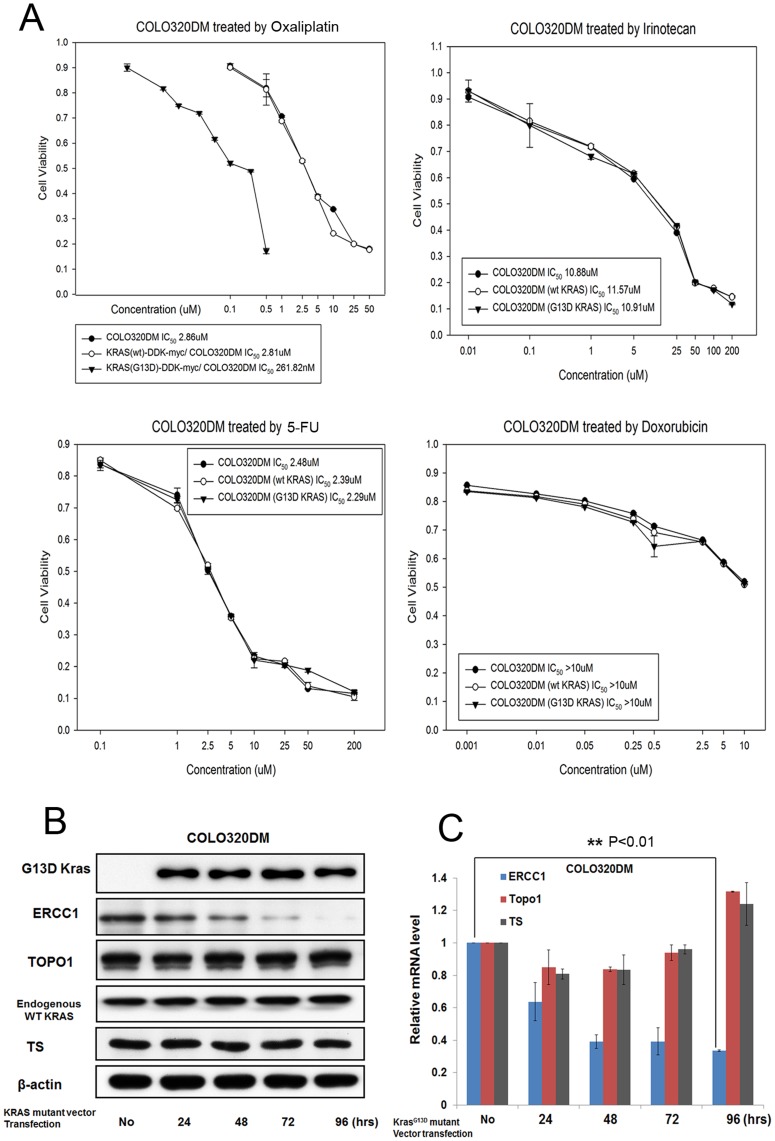
Overexpressing KRAS in KRAS wild-type CRC cells leads to oxaliplatin sensitivity and ERCC1 downregulation. (A) KRAS^G13D^-DDK-myc-COLO320DM cells were more sensitive to oxaliplatin, but have the same sensitivity to irinotecan, 5FU, and doxorubicin than parental COLO320DM cells, as demonstrated by MTT assay. (B) The protein level of ERCC1, but not those of TOPO1 or TS, was downregulated after COLO320DM cells were transfected by the KRAS^G13D^ mutant vector. (C) The mRNA level of ERCC1, but not those of TOPO1 or TS, was downregulated after COLO320DM cells were transfected by the KRAS^G13D^ mutant vector. **: *p*<0.01.

### Knocking-down of KRAS and ERCC1

Two types of both KRAS and ERCC1 small interfering RNAs (siRNA) and scrambled nonspecific (negative control) siRNA were purchased from Applied Biosystems, Inc. (Foster City, CA, USA). For KRAS gene knockdown, DLD-1^G13D^ and SW480^G12V^ cells were first transfected with KRAS- or scrambled siRNAs for 1 day using the Lipofectamine2000 transfection reagent (Invitrogen, Carlsbad, CA, USA) according to the manufacturer's instructions. The transfected cells were then treated with oxaliplatin, irinotecan, 5FU and doxorubicin with various concentrations for the following 72 hours. The protein lysate and mRNA of parental and KRAS knockdown DLD-1^G13D^ and SW480^G12V^ cells were collected in 24, 48, 72 and 96 hours after transfection for evaluation of KRAS knockdown magnitude by western blot. For ERCC1 gene knockdown, COLO320DM cells transfected with two different ERCC1- or scrambled SiRNAs were treated with oxaliplatin for 72 hours. The protein lysate and mRNA of parental and ERCC-knocked-down COLO320DM cells were collected in 24, 48, 72 and 96 hours post-transfection for evaluating the ERCC1 knockdown effect by western blot and qRT-PCR.

**Figure 4:**
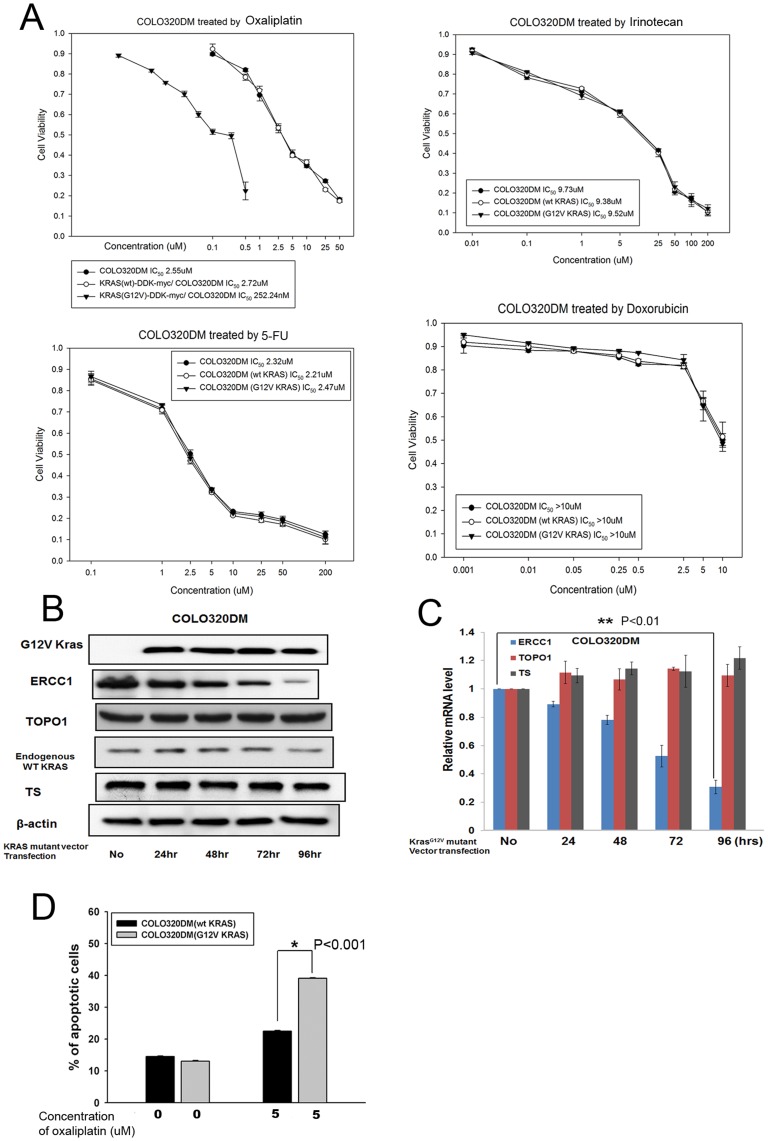
Overexpressing KRAS by another KRAS overexpression vector (G12V) in KRAS wild-type CRC cells leads to oxaliplatin sensitivity and ERCC1 downregulation. (A) KRAS^G12V^-DDK-myc-COLO320DM cells were more sensitive to oxaliplatin, but have the same sensitivity to irinotecan, 5FU, and doxorubicin than parental COLO320DM cells, as demonstrated by MTT assay. (B) The protein level of ERCC1, but not those of TOPO1 or TS, was downregulated after COLO320DM cells were transfected by the KRAS^G12V^ mutant vector. (C) The mRNA level of ERCC1, but not those of TOPO1 or TS, was downregulated after COLO320DM cells were transfected by the KRAS^G12V^ mutant vector. **: *p*<0.01. (D) Increased percentage of apoptosis, from 22.5%±0.2% to 39.1%±0.2% of apoptosis (P<0.001), has been demonstrated when KRAS^wt^-DDK-myc-COLO320DM cells, were compared to KRAS^G12V^-DDK-myc-COLO320DM cells, in which, both were treated by the same concentration of oxaliplatin in 5 µM. *: *p*<0.001.

### Overexpression of KRAS and ERCC1

The pCMV6-Myc-DDK-tagged-KRAS vector was purchased from OriGene Technologies (Rockville, MD, USA). DNA-sequence-encoding KRAS G12V and G13D mutation were generated by site-directed mutagenesis and cloned into the pCMV6-Myc-DDK-tagged-KRAS vector. The sequences of KRAS G12V and G13D mutation were as follows: 5′-GTTGTGGTAGTTGGAGCT**GTT**GGCGTAGGCAAGAATGCC-3′; reverse: 5′-GGCACTCTTGCCTACGCCAACAGCTCCAACTACCACAAG-3′ and forward: 5′-GGTAGTTGGAGCTGGT**GAC**GTAGGCAAGAGTGCC-3′; reverse: 5′-GGCACTCTTGCCTACGTCACCAGCTCCAACTACC-3′, respectively. For KRAS overexpression, COLO320DM cells were transiently transfected with the pCMV6-Myc-DDK-tagged-KRAS, -KRAS^G12V^, and -KRAS^G13D^ vectors. After 24-hour of transfection, cells were treated with oxaliplatin, irinotecan, 5FU, and doxorubicin with various concentrations for the following 72 hours. The protein lysate and mRNA of COLO320DM cells transfected by the pCMV6-Myc-DDK-tagged-KRAS, -KRAS^G12V^, and -KRAS^G13D^ vectors were collected at 24, 48, 72, and 96 hours for evaluation of KRAS overexpression magnitude by western blot. For ERCC1 overexpression, SW480^G12V^ cells were transfected by the pCMV6-ERCC1-GFP vector (OriGene Technologies, Rockville, MD, USA) for 24 hours, and treated with oxaliplatin for 72 hours. The protein lysate of SW480^G12V^ cells transfected by the ERCC1-GFP vector was collected at 24, 48, 72, and 96 hours for evaluation of ERCC1 overexpression magnitude by western blot.

**Figure 5:**
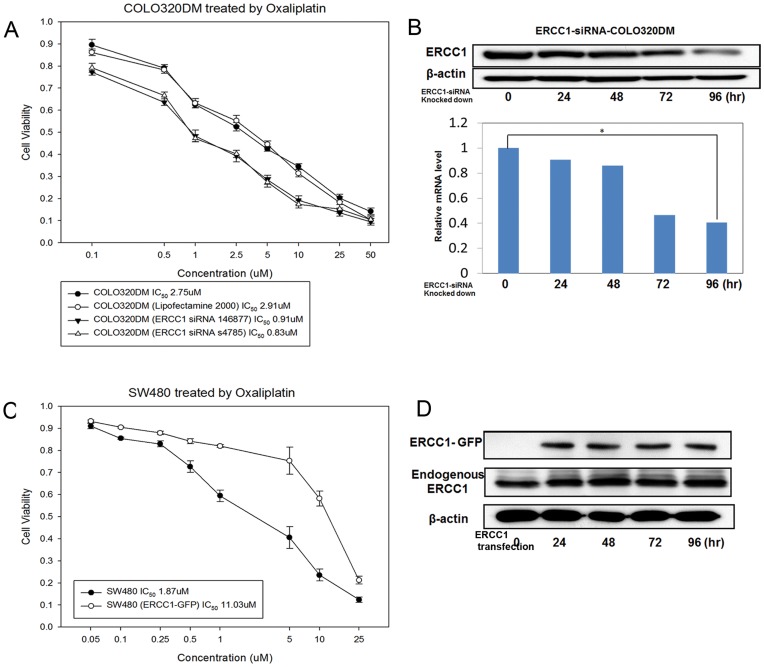
Validating ERCC1 as the predictor of oxaliplatin sensitivity in CRC cells. (A) ERCC1-knocked-down COLO320DM cells were more sensitive to oxaliplatin than parental COLO320DM cells, as demonstrated by MTT assay. (B) Protein and mRNA levels of ERCC1 were downregulated when COLO320DM cells were knocked-down by ERCC1 siRNA. *: *p*<0.05 (C) ERCC1-GFP-SW480^G12V^ cells were more resistant to oxaliplatin than parental SW480^G12V^ cells. (D) Ectopic ERCC1 was upregulated after SW480^G12V^ cells were transfected by the ERCC1-GFP expression vector.

### Cell Viability and Apoptotic Analyses

Cell viability was assessed by using the 3-(4,5-dimethylthiazol-2-yl)-2,5-diphenyltetrazolium bromide (MTT; Tokyo Chemical Industry Inc., Tokyo, Japan) assay in 6 replicates. Initially, COLO320DM, SW480^G12V^, and DLD-1^G13D^ cells were seeded at 3500, 4500, and 3000 cells per well in 96-well flat-bottomed plates, respectively. After 24-hour incubation, SW480^G12V^ and DLD-1^G13D^ cells were transfected by KRAS- and scrambled siRNAs, and COLO320DM cells were transfected by the pCMV6-Myc-DDK-tagged KRAS, -KRAS^G12V^, and -KRAS^G13D^ vectors, as described. After KRAS-siRNAs were transfected to DLD-1^G13D^/SW480^G12V^ cells and KRAS-mutant vectors to COLO320DM cells for 24 hours, cells were treated with oxaliplatin, irinotecan, 5FU, and doxorubicin at various concentrations in 10% FBS-supplemented RPMI-1640 for 72 hours. The control cells were mixed with DMSO at a concentration equal to that in drug-treated cells. Cell viability of these treated cells was measured by adding 200 µl of 0.5 mg/ml MTT solubilized in DMSO to each well, and cells were incubated in the CO_2_ incubator at 37°C for 2 hours after removal of the medium. Absorbance was determined at 570 nm. Concentrations of compounds that inhibited viability by 50% (IC_50_) were determined using the median effect method by employing CalcuSyn software (Biosoft, Ferguson, MO, USA).

**Figure 6:**
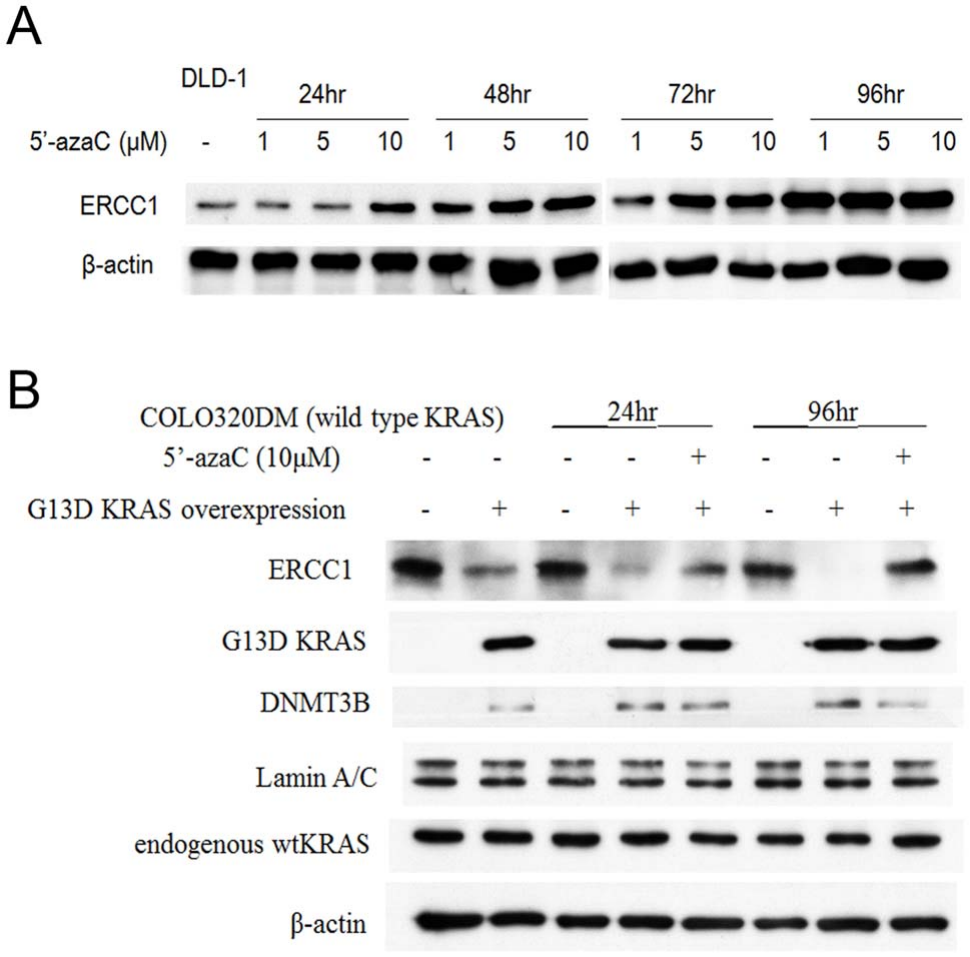
Downregulation of ERCC1 expression in KRAS-mutant CRC cells might be related to hypermethylation of ERCC1gene, which possibly induced by up-regulation of DNMT3B (DNA methyltransferase 3B). (A) Protein expression of ERCC1 in DLD-1(KRAS^G13D^ mutation) cells is up-regulated after 5′-azacitidine (de-methylating agent) treatment for 96 hours, which implied that the downregulation of ERCC1 in KRAS-mutant CRC cells might be partly through ERCC1 hypermethylation. (B) Downregulation of ERCC1 in COLO320DM (KRAS wild-type) cells transfected by KRAS^G13D^-mutant-vector for 24 and 96 hours may not only be restored by 5′-azacitidine in 10 µM, but also caused up-regulation of DNMT3B.

The fraction of apoptotic cells, after KRAS overexpressed in COLO320DM cells, and treated by oxaliplatin, was assessed by flow cytometry with Annexin V-FITC. COLO320DM cells were seeded at 2.5×10^5^ cells/per well for scrambled and KRAS^G12V^-mutant-vector transfection in 6-well plates. After 6 hours of transfection, transfection medium was replaced by the regular medium. Oxaliplatin with the concentration of 5 µM was given to transfected COLO320DM cells in the next day. Transfected COLO320DM cells were then trypsinized and collected for analysis after 48 hours of oxaliplatin treatment. Cells were centrifuged at 300 g for 5 minutes at room temperature, and the cell suspension was stained with Annexin V-FITC (Annexin V assay kit, BD Biosciences Pharmingen) and propidium iodide at room temperature for at least 15 minutes in the dark. The cells were then analyzed by FACScan flow cytometer and Cell Quest program. The proportion of apoptotic cells was the proportion of cells stained with Annexin V-FITC.

### Western Blot Analysis

KRAS-overexpressed COLO320DM and KRAS-knocked-down SW480^G12V^ and DLD-1^G13D^ cells treated with various concentrations of oxaliplatin, irinotecan, and 5FU for 72 hours in 6-cm dishes (1×10^5^ cells per dish) were collected and lysed with a RIPA lysis buffer [50 mmol/L Tris-HCl (pH 8.0), 150 mmol/L NaCl, 1% NP40, 0.5% sodium deoxycholate, 0.1% SDS] (Sigma Cat. No. R0278). Protein concentrations of the lysate were determined using a Pierce BCA protein assay kit (Thermal Scientific, Odessa, Texas, USA). Equivalent amounts of protein from each lysate were subjected to SDS-PAGE and then transferred to nitrocellulose membranes for immunoblotting. The transblotted membranes were washed twice with Tris-buffered saline (TBS) containing 0.1% Tween 20 (TBST). After blocking with TBST containing 5% nonfat milk for 40 minutes, the membranes were incubated with the appropriate primary antibody in TBST containing 1% nonfat milk at 4°C overnight. All of the primary antibodies were diluted in an appropriate concentration of 1% nonfat milk-containing TBST. After treatment with the primary antibody, the membranes were washed twice with TBST for 20 minutes, followed by goat anti-rabbit or anti-mouse IgG-horseradish peroxidase–conjugated secondary antibody (diluted 1∶3000) for 1 hour at room temperature and washed 3 times with TBST for 1 hour. The membranes were developed using an enhanced chemiluminescence horseradish peroxidase substrate (Millipore, Bedford, MA, USA) according to the manufacturer's instructions.

### Real-time Quantitative Reverse Transcriptase Polymerase Chain Reaction (qRT-PCR)

KRAS-overexpressed COLO320DM, KRAS-knocked-down SW480^G12V^ and DLD-1^G13D^ cells treated with various concentrations of oxaliplatin, irinotecan, and 5FU for 24, 48, and 72 hours, respectively, were collected and lysed in a Trizol reagent (Invitrogen, Carlsbad, CA, USA) and stored at −20°C. The RNA of these cells was extracted according to the manufacturer's instructions. cDNAs were synthesized from total RNA (1 µg) using the Applied Biosystems High-Capacity cDNA Archive kit according to the manufacturer's instructions. The cDNAs from 50-ng total RNA were quantified using the Taqman Universal or SYBR Green PCR Master Mix (Applied Biosystems, Foster City, CA, USA) on an ABI PRISM 7900 Sequence Detection System (Perkin-Elmer/Applied Biosystems). The primer sequences of ERCC1 (ABI Taqman assay ID: Hs01012158_ml), TOPO I (ABI Taqman assay ID: Hs00243257_ml), TS (ABI Taqman assay ID: Hs00426586_ml), and β-actin gene (ABI Taqman assay ID: Hs99999903_ml) as an endogenous control were all purchased from Applied Biosystems (Foster City, CA, USA). Conditions for PCR were 50°C for 2 minutes, 95°C for 10 minutes, and 40 cycles of 95°C for 15 seconds (denaturation) and 60°C for 1 minute (annealing/extension). The relative mRNA amount of the target gene/endogenous control gene (β-actin) was calculated using the ΔCt (threshold cycle) method, as follows: relative expression = 2-ΔCt, where ΔCt = Ct (target gene) - Ct (β-actin).

### Statistical Analysis

For cell line studies, all data were repeated for at least 3 independent experiments. Quantitative data are represented as mean ± SD. Comparisons between data within the same experiments were analyzed using the Student's *t* test. A *p*-value of <0.05 was considered statistically significant.

## Results

### Knocking-down KRAS in KRAS-mutant CRC Cells Increases Oxaliplatin Resistance and Causes ERCC1 Overexpression

Knocking-down KRAS in DLD-1^G13D^ cells resulted in cells more resistant to oxaliplatin, but not to irinotecan, and 5FU, standard chemotherapeutic agents for CRC, and not to doxorubicin, broad spectrum chemotherapeutic agent for other major cancers. Two different KRAS-siRNAs were transfected to DLD-1^G13D^ cells. The IC_50_ of the first-paired parental DLD-1^G13D^/KRAS-siRNA(1)-DLD-1^G13D^ cells treated with oxaliplatin for 72 hours was 3.97/33.07 µM. The second-paired parental DLD-1^G13D^/KRAS-siRNA(2)-DLD-1^G13D^ cells was 3.97/13.49 µM. The IC_50_ of paired parental DLD-1^G13D^/KRAS-siRNA-DLD-1^G13D^ cells treated with irinotecan, 5FU and doxorubicin remained unchanged (Figure 1A). ERCC1, TOPO I and TS, which were thought to be biomarkers for predicting the sensitivity of oxaliplatin [1], irinotecan [10] and 5FU [2], respectively, were further checked by western blot and qRT-PCR (Figures 1B, and1C) to explore mechanisms behind our findings. Only ERCC1 expression was upregulated after KRAS knockdown; in contrast, TOPO I and TS remained constant both in protein and mRNA levels. KRAS knockdown efficiency was evaluated by western blot, which showed diminished of KRAS expression after 72-hour of knocking-down the KRAS gene (Figure 1B).

To further consolidate our observation, we used another CRC cell, SW480, which harbored another KRAS mutant subtype, G12V, to repeat the same experimental procedures. In summary, parental SW480^G12V^ cells was, as expected, more sensitive to oxaliplatin than KRAS knocked-down SW480^G12V^ cells. The IC_50_ of parental SW480^G12V^/KRAS-siRNA-SW480^G12V^ cells treated by oxaliplatin for 72 hours was 2.08/13.53 µM, but that of parental SW480^G12V^/KRAS-siRNA-SW480^G12V^ cells treated with irinotecan, 5FU, and doxorubicin remained unchanged (Figure 2A). ERCC1, TOPO I, and TS were simultaneously checked by western blot and qRT-PCR (Figures 2B and 2C), and again only ERCC1 was upregulated after KRAS knockdown, with TOPO I and TS levels remaining unchanged in protein and mRNA levels. Similarly, KRAS knockdown efficiency was measured by western blot, which showed a decline in KRAS expression after 72-hour of knocking-down the KRAS gene (Figure 2B).

### Overexpressing KRAS in KRAS Wild-type CRC Cells Leads to Oxaliplatin Sensitivity and ERCC1 Downregulation

Overexpression of KRAS in COLO320DM cells by KRAS-mutant vectors resulted in cells more sensitive to oxaliplatin. The IC_50_ of parental COLO320DM/KRAS^G13D^-DDK-myc-COLO320DM cells treated by oxaliplatin for 72 hours was 2.86/0.26 µM, but that of parental COLO320DM/KRAS^G13D^-DDK-myc-COLO320DM cells treated with irinotecan, 5FU, and doxorubicin remained unchanged (Figure 3A). ERCC1, TOPO I, and TS were checked by western blot and qRT-PCR (Figures 3B and 3C), which showed that only ERCC1 was downregulated without any change in protein and mRNA levels in TOPO I and TS after KRAS overexpression. The expression of ectopic KRAS and endogenous KRAS was measured by western blot, which showed a robust expression of ectopic KRAS, with a constant expression of endogenous KRAS after 24-hour overexpression of the KRAS gene (Figure 3B).

The same results were also found in COLO320DM cells transfected with the KRAS^G12V^-mutant vector. The IC_50_ of parental COLO320DM/KRAS^G12V^-DDK-myc-COLO320DM cells treated with oxaliplatin for 72 hours was 2.55/0.25 µM, but that of parental COLO320DM/KRAS^G12V^-DDK-myc-COLO320DM cells treated with irinotecan, 5FU, and doxorubicin remained unchanged (Figure 4A). Again, only ERCC1 was downregulated without any change of TOPO I and TS in protein (Figure 4B) and mRNA levels (Figure 4C) after KRAS overexpression. The expression of ectopic KRAS and endogenous KRAS after 24-hour overexpression of the KRAS gene is shown in Figure 4B. To further strengthen the finding that KRAS^G12V^-DDK-myc-COLO320DM cells were more sensitive to oxaliplatin than parental COLO320DM cells, flow cytometry with annexin V-FITC was performed. Consequently, increased percentage of apoptosis, from 22.5%±0.2% to 39.1%±0.2% of apoptosis (P<0.001), has been found when parental COLO320DM cells, transfected by KRAS^wt^-DDK-myc-vector, were compared to COLO320DM cells, transfected by KRAS^G12V^-DDK-myc-vector, in which, both were treated by the same concentration of oxaliplatin in 5 µM (Figure 4D).

### Validating ERCC1 Expression as the Predictor of Oxaliplatin Sensitivity

#### Knocking-down ERCC1 in KRAS wild-type CRC cells restores oxaliplatin sensitivity

To further confirm the relationship between ERCC1 expression and oxaliplatin sensitivity, we knocked-down the ERCC1 gene using 2 different ERCC1-siRNAs in KRAS-wild-type cells (COLO320DM). We found that the IC_50_ of the first-paired parental COLO320DM/ERCC1-siRNA(1)-COLO320DM cells treated with oxaliplatin for 72 hours was 2.75/0.91 µM (Figure 5A). The second-paired parental COLO320DM/ERCC1-siRNA(2)-COLO320DM cells was 2.75/0.83 µM (Figure 5A). The protein and mRNA expression levels of ERCC1 were downregulated after ERCC1 was knocked-down by ERCC1-siRNA in COLO320DM cells (Figure 5B).

#### Overexpressing ERCC1 in KRAS-mutant CRC cells causes oxaliplatin resistance

Overexpression of ERCC1 in SW480^G12V^ cells by the ERCC1-overexpressing vector caused SW480^G12V^ cells to become more resistant to oxaliplatin. The IC_50_ of parental SW480^G12V^/ERCC1-GFP-SW480^G12V^ cells treated with oxaliplatin for 72 hours was 1.87/11.03 µM (Figure 5C). Western blot was used to determine the ERCC1-GFP overexpression level after transfection (Figure 5D).

## Discussion

Our study shows that KRAS mutation is a predictor of oxaliplatin sensitivity in colon cancer cells by ERCC1 downregulation. This may provide an important step to personalized chemotherapy in colon cancer.

In the pre-targeted therapy era, Tournigand et al [11] published the pivotal article indicating that, first-line chemotherapy with either irinotecan/5FU/lecovorin (FOLFIRI) or oxaliplatin/5FU/leucovorin (FOLFOX6) in “non-selected” metastatic CRC patients, sequentially followed by the other after progression, did not influence overall survival (OS). Their article also indicated that both regimens may be recommended as a first-line treatment for advanced CRC. In the modern era of targeted therapy, current treatment has been advanced to personalized therapy after the wild-type KRAS gene was identified as a predictor for the EGFR monoclonal antibody. KRAS status has been recommended to be routinely checked in daily oncological practice. To identify better predictors in current chemotherapy or newer treatment targets for KRAS-mutant CRC patients is warranted. Our study was initiated to find better predictors in current chemotherapy, for which the hypothesis was generated from subgroup analyses of randomized prospective clinical trials, PRIME [8] and OPUS [7], versus CRYSTAL [9]. According to our findings, KRAS-mutant CRC patients might benefit more from receiving first-line oxaliplatin-based regimens than KRAS-wild-type patients. This phenomenon warrants further confirmation by large prospective clinical trials.

Our data demonstrated that KRAS mutation in CRC cells caused ERCC1 downregulation. This significant finding might imply that some other unknown druggable targets may still be responsible for KRAS-mutant CRC treatment in addition to the traditional RAS/RAF/MEK/ERK pathway. To explore these unknown targets, studies designed from epigenetic and/or genetic point of views may be helpful. From epigenetic point of view, hypermethylation causes gene silencing is well-known [12], [13]. In our study, we have found that the protein expression of ERCC1 in DLD-1(KRAS^G13D^ mutation) cells is up-regulated after 5′-azacitidine (de-methylating agent) treatment for 96 hours (Figure 6A), which indicated that the downregulation of ERCC1 in KRAS-mutant CRC cells might be partly through ERCC1 hypermethylation. We also found that the downregulation of protein expression of ERCC1 in COLO320DM (KRAS wild-type) cells transfected by KRAS-mutant-vector for 24 and 96 hours may be restored by 5′-azacitidine (Figure 6B). This further implied that the downregulation of ERCC1 expression in CRC cells is not only partly through hypermethylation, but also determined by the changes of KRAS expression in CRC cells. Because downregulation of ERCC1 in COLO320DM cells, transfected by KRAS^G13D^-mutant-vector, might be caused by hypermethylation of ERCC1, we further checked DNMT3B (DNA methyltransferase 3B), whose major role is to proceed the process of methylation. We found that DNMT3B was upregulated when ERCC1 was dowenregulated in COLO320DM cells, transfected by KRAS^G13D^-mutant-vector (Figure 6B). DNMT3B may again suppress by 5′-azacitidine, which depicted that DNMT3B is probably responsible for the methylation process. Therefore, our data showed that ERCC1 downregulation in KRAS-mutant CRC cells might be through ERCC1 hypermethylation. We proposed that KRAS-mutant CRC cells might have higher methylation rate on CpG islands of ERCC1 promoter region than KRAS-mutant cells transfected by KRAS-siRNA. This hypothesis may be validated by comparing the possible differences of hypermethylation on ERCC1 promoter region between KRAS-mutant cells and KRAS-mutant cells transfected by KRAS-siRNA by methylation specific PCR and/or sodium bisulfite sequencing analysis [14]. The whole concept would be that KRAS activating mutation might cause DNMT3B upregulation. Subsequently, DNMT3B might bind to the promoter region of ERCC1 to result in hypermethylation of ERCC1 gene. Finally, hypermethylation of ERCC1 gene results in downregulation of ERCC1 expression. Alternatively, from genetic point of view, as KRAS mutation is an activating mutation, which has been widely accepted [15], [16], we proposed that there might be an existed unknown factor, which may be inhibited by the activation of KRAS gene or its downstream signals. This factor needs also to be an activating factor to activate ERCC1 gene. Then, once KRAS gene is mutated (activated), this factor might be inhibited, and the amount of this factor might be declined. Subsequently, the expression of ERCC1 might be suppressed due to the lack of this factor. To conduct this kind of studies, reporter gene constructs using ERCC1 promoter, luciferase activity assay and chromatin immunoprecipitation may be thus needed [17].

Although the detailed mechanisms behind these findings remain elusive, crosstalks between the mutated KRAS gene and DNA repair machinery pathways, which might also be responsible for the effect of oxaliplatin-based treatment, have been investigated [18], [19]. Additionally, various new generations of microarray-based technologies, comparing same given cells with/without KRAS mutation by knocking-down and overexpressing the KRAS gene accordingly, may be another helpful way in defining new targets for KRAS-mutant CRC treatment [5], [6].

Overexpression of ERCC1 is associated to the resistance to platinum-based chemotherapy [1], [20], [21], [22], [23], [24], which has been demonstrated in various kinds of cancers, including esophageal cancers [25], non-small cell lung cancers [26], and bladder cancers [27]. These findings are also compatible to the current study. In our study, we demonstrated that KRAS wild-type (COLO320DM) CRC cells were more resistant to oxaliplatin than the same given cells (COLO320DM) transfected by KRAS-mutant-vectors. We also demonstrated one of the reasons for the resistance may be related to higher ERCC1 expression in parental COLO320DM cells compared to COLO320DM cells transfected by KRAS-mutant-vectors.

Our in vitro experiments had limitations. First, 2 of 7 KRAS-mutant subtypes, which represented 40% of total KRAS mutation [28], were chosen as models in our study; this might not represent the biological behaviors of all KRAS-mutant subtypes in CRC cells. Further comprehensive studies with all KRAS mutant subtypes may be warranted. Second, mechanisms behind ERCC1 downregulation caused by KRAS mutation remain elusive. Although we proposed two possible ways to approach this issue, deeper understanding the biology of KRAS gene and the crosstalk between KRAS gene and DNA repair machinery may facilitate the advances on this issue.

In conclusion, our data suggested that KRAS mutation is a predictor of oxaliplatin sensitivity in colon cancer cells by ERCC1 downregulation.
